# Clinical and radiologic features of soft tissue sarcoma in trunk and extremities that underwent unplanned excision

**DOI:** 10.1371/journal.pone.0311300

**Published:** 2024-12-05

**Authors:** Yujin Seo, Young Cheol Yoon, Hyun Su Kim, Sung Wook Seo, Yoon-La Choi, Kyunga Kim, Ji Hyun Lee

**Affiliations:** 1 Department of Radiology and Center for Imaging Science, Samsung Medical Center, Sungkyunkwan University School of Medicine, Seoul, Republic of Korea; 2 Department of Orthopedic Surgery, Samsung Medical Center, Sungkyunkwan University School of Medicine, Seoul, Republic of Korea; 3 Department of Pathology and Translational Genomics, Samsung Medical Center, Sungkyunkwan University School of Medicine, Seoul, Republic of Korea; 4 Biomedical Statistics Center, Research Institute for Future Medicine, Samsung Medical Center, Seoul, Republic of Korea; University of Texas M. D. Anderson Cancer Center, UNITED STATES OF AMERICA

## Abstract

**Background:**

To identify the clinical and imaging characteristics of soft tissue sarcomas (STS) of the trunk and extremities that undergo unplanned excision.

**Methods:**

This retrospective study evaluated the data of patients with STS in the trunk or extremities between January 2008 and December 2021. Patients were divided into unplanned and planned excision groups based on their initial treatment. The distribution of histologic subtypes was analyzed, and the magnetic resonance imaging features were evaluated. Multivariable logistic regression was performed to identify variables independently associated with unplanned excision.

**Results:**

A total of 305 patients (mean age±standard deviation, 51±16.4 years; 179 males) were analyzed. The most prevalent subtype in the unplanned excision group was myxofibrosarcoma (22.3%). The unplanned excision group had a significantly smaller tumor size (p < 0.001) and more frequent superficial (p < 0.001) locations. Lobulated shape and peritumoral abnormalities were present in 70.0% and 50.0% of the unplanned excision group, respectively. Tumor size (adjusted odds ratio [OR], 0.87 per 1 cm increase; 95% confidence interval [CI], 0.77–0.98; p = 0.025) and superficial location (adjusted OR, 3.48; 95% CI, 1.57–7.72; p = 0.002) were independently associated with unplanned excision.

**Conclusion:**

Myxofibrosarcoma is associated with a high frequency of unplanned excision. A significant number of patients in the unplanned excision group demonstrated a lobulated shape and peritumoral abnormalities. Only small tumor size and superficial location were independently associated with unplanned STS excision.

## Introduction

Soft tissue sarcomas (STS), a heterogeneous group of malignant tumors accounting for approximately 1% of all adult malignancies, encompass over 100 different histological and molecular subtypes [1]. The standard surgical procedure for localized STS is a wide excision with negative margins [2]. However, due to their rarity and heterogeneity, STS are often unsuspected and misdiagnosed as benign tumors, leading to unplanned excision where tumors are excised without adequate consideration of tumor-free margins [3, 4]. Tumor bed excision to remove potential residual STS and contaminated tissues is considered standard practice after unplanned STS excision [4, 5].

Unplanned excision is associated with higher risk of local recurrence despite full further oncological management [5–8] and tumor bed excision after unplanned excision is more extensive than planned excision [4, 9, 10], leading to significant morbidity. Thus, avoiding such unfavorable operations is crucial to improve the prognosis of patients with STS. However, unplanned excision is still the primary surgical intervention in 36.5–53.3% of patients [8, 11]. Although several studies have investigated the clinical characteristics and prognosis of STS that undergo unplanned excision [8, 10, 12–15], few have focused on their histologic and imaging characteristics [13, 14].

Magnetic resonance imaging (MRI) remains the preferred imaging modality for local staging, characterization, and preoperative planning of STS [2, 16]. Despite efforts to improve diagnostic accuracy in discriminating STS from benign soft tissue lesions, the significant overlap of MRI features between the two entities continue to pose a challenge [17–21]. Although several useful MRI features for diagnosing STS have been reported, such as large size, deep location, and heterogeneous signal intensity [19–21], there is a lack of studies on the imaging features of STS that underwent unplanned excision. Exploring such imaging findings would be beneficial as they may help surgeons and radiologists in realizing the potential risk of unplanned excision and working toward reducing its incidence.

Thus, we aimed to identify the clinical characteristics, including histologic subtypes and imaging features, of STS of the trunk and extremities that underwent unplanned excision.

## Materials and methods

The institutional review board of Samsung Medical Center approved this retrospective study and waived the requirement for patient consent to use the clinical data (IRB file no. 2023-01-008). The study was conducted under the principles of the Declaration of Helsinki.

### Study population

We retrospectively analyzed data from 477 consecutive adult patients (≥18 years) histologically diagnosed with STS of the trunk or extremities at our hospital between January 2008 and December 2021. Only patients initially diagnosed with STS were included, and those with recurrent tumors from previously treated STS were excluded (n = 85). Patients with well-differentiated liposarcoma (n = 36) were also excluded given their distinct radiologic findings and diagnostic algorithms, including *MDM2* gene amplification [22]. We also excluded patients with tumors categorized as intermediate according to the 2020 WHO classification of soft tissue tumors [23] (n = 20).

**Fig 1:**
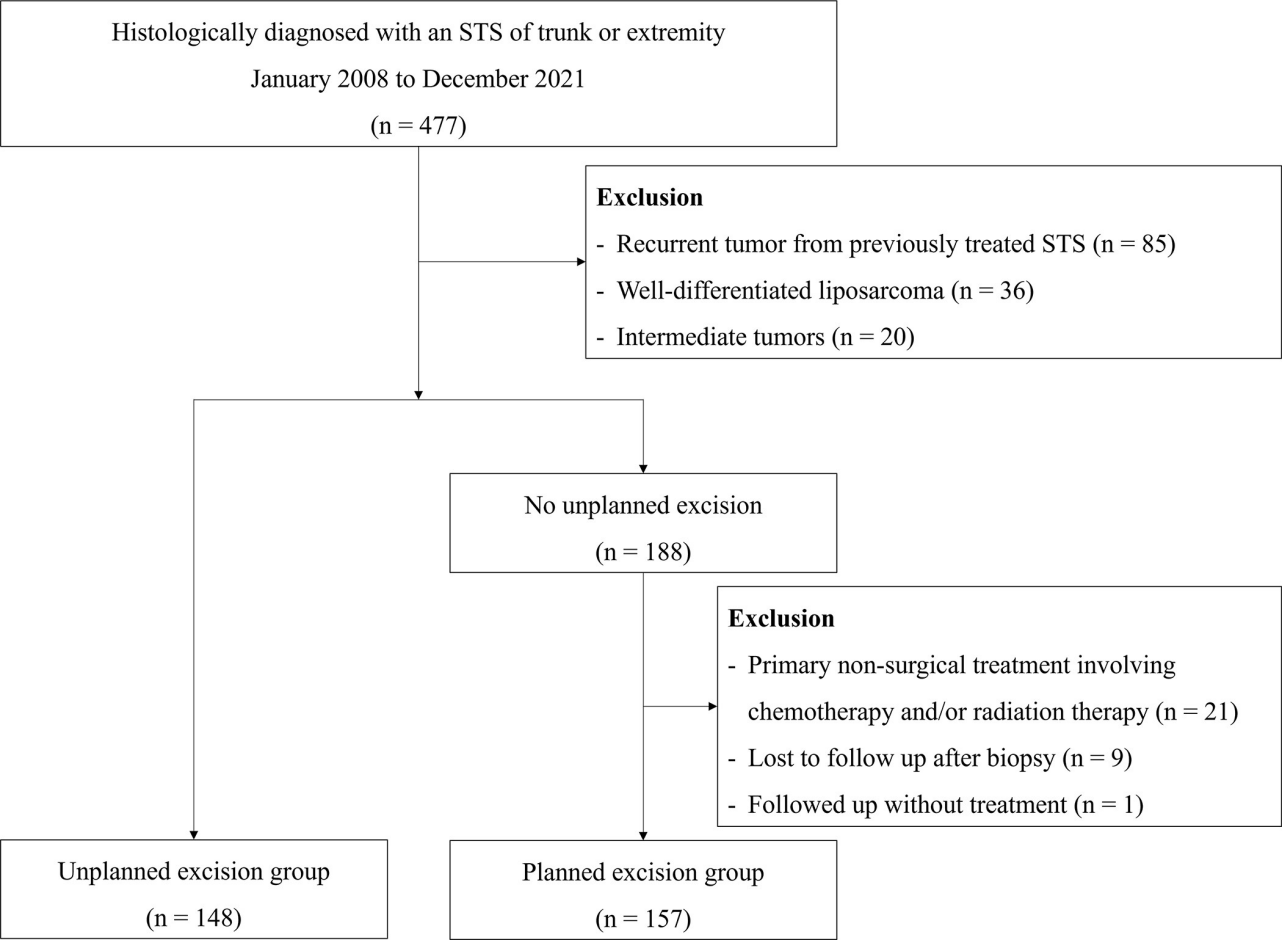
Flow chart of patient selection. *STS* soft tissue sarcoma.

Patients were divided into two groups according to their initial treatment. The unplanned excision group consisted of patients who underwent unplanned excision, defined as gross tumor removal without consideration of preoperative imaging or the necessity of tumor removal with an adequate margin of normal tissue [3, 24]. Among the patients who did not undergo unplanned excision, those who underwent primary planned excision were categorized into the planned excision group. Patients who received primary nonsurgical treatment involving chemotherapy and/or radiation therapy (n = 21), lost to follow-up after biopsy (n = 9), and who were followed-up without receiving any treatment (n = 1) were excluded (Fig 1).

### Clinical and pathological data

The data for this study were obtained from the Clinical Data Warehouse Darwin-C (CDW) of the Samsung Medical Center and by reviewing the electronic medical records. The collected variables included age at diagnosis, sex, tumor site (trunk, upper extremity, or lower extremity), histological diagnosis, and treatment. In the unplanned excision group, the final histological diagnosis of patients who underwent re-excision relied on the re-excision surgical specimen. If no residual tumor was present, or if re-excision was not performed, the initial histopathological slides from the unplanned excision were reviewed for histological diagnosis. In the planned excision group, the final histological diagnosis was based on the surgical specimens. If no residual tumor was detected in the surgical specimen after neoadjuvant therapy, a final histological diagnosis was made using preoperative biopsy specimens obtained via core needle or incisional biopsy. A pathologist with experience in musculoskeletal sarcoma (Y.-L.C.) reviewed all slides.

### Image analysis

MRI equipment and protocols, such as field of view, matrix size, slice thickness, and echo train length, varied across institutions and depend on the anatomical position being imaged.

Image analysis of pre-treatment MRI was done by a board-certified radiologist with 8 years of experience in musculoskeletal radiology (J.H.L.), who was blinded to patient information. The following MRI findings were evaluated: (1) size, measured as the maximum diameter of the lesion irrespective of plane; (2) superficial location, defined as superficial regarding the deep peripheral fascia with or without contacting it but not penetrating through it [25]; (3) lobulated shape, determined present when the tumor margin showed two or more projections; (4) peritumoral abnormality, considered present when the tumor showed peritumoral enhancement/edema, defined as presence of infiltrative areas of post-contrast enhancement and/or high signal intensity on T2-weighted images that showed low-to-intermediate signal intensity on T1-weighted images, or when indistinct tumor margin is noted; (5) hemorrhage, deemed present when there were foci of high signal intensity on T1-weighted images that did not show signal drop on fat-suppressed images [17]; (6) necrosis, considered present when a fluid-like signal with an irregular margin and rim enhancement was detected, without contrast enhancement of the necrotic fluid [17]; (7) signal intensity heterogeneity, defined as ≥50% of the tumor volume showed areas of low, intermediate, and high signal intensity in either T1-weighted or T2-weighted images; (8) cyst-like appearance, considered present when the majority of the lesion showed signal intensity similar to water with bright on T2-weighted image and hypointense on T1-weighted image [26]; (9) bone involvement, defined as change in cortical and medullary signal intensity and/or cortical destruction [27]; (10) neurovascular bundle encasement, defined as tumor abutment of the nerve or vessel exceeding 180 degrees of the circumference [28] (S1–S4 Figs). The quality of all MRIs was assessed to exclude those with suboptimal quality for analyses.

### Statistical analysis

Descriptive statistics were used to summarize the patient characteristics. The distribution of the 10 most prevalent histological subtypes, excluding unspecified sarcomas, was analyzed in both groups. A one-sample proportion test was used to assess whether the frequency of unplanned excisions for each histological subtype differed from the overall frequency of unplanned excisions.

The clinical and MRI features of the patients were compared between the two groups. Categorical variables were evaluated using the chi-square or Fisher’s exact test, whereas continuous variables were analyzed using the two-sample t- or Mann-Whitney U test. Multivariable logistic regression analysis was conducted to identify variables independently associated with unplanned excision of STS, including variables that showed significant differences between the two groups in the between-group analysis.

P < 0.05 was considered statistically significant. All statistical analyses were performed using the Rex-Pro software (version 3.6.3; Rexsoft, Co. Ltd., Seoul, Republic of Korea).

## Results

A total of 305 patients (179 males, 126 females) with a mean age of 51 years (standard deviation: 16.4) were included in the analyses. The unplanned excision group comprised 148 patients (48.5%) who were referred to our hospital after unplanned excision at an outside hospital (n = 146) or underwent unplanned excision at a nonspecialized department in our hospital (n = 2). In the unplanned excision group, 125 patients underwent re-excision, whereas 12 received adjuvant chemotherapy and/or radiation therapy without re-excision. Eleven patients were followed-up without subsequent treatment. The planned excision group comprised 157 patients, including 148 patients who received a preoperative diagnosis through biopsy, and 9 patients who underwent immediate wide excision based on a high suspicion of malignancy in imaging studies.

Pre-treatment MRI was available for 195 patients (63.9%), including 40 patients in the unplanned excision group and 155 patients in the planned excision group. The quality of all pre-treatment MRI was acceptable for analysis. All MRI features, except for hemorrhage (n = 3), for which T1-weighted images were not available, and necrosis (n = 12), for which only noncontrast images were obtained, were evaluated.

### Histological diagnosis and distribution of each histologic subtypes

In the unplanned excision group, the final histological diagnosis was based on the re-excision specimens from 70 patients and the initial unplanned excision specimens from 78 patients. In the planned excision group, 156 patients were diagnosed based on surgical specimens, whereas 1 patient was diagnosed based on preoperative biopsy specimens.

**Fig 2:**
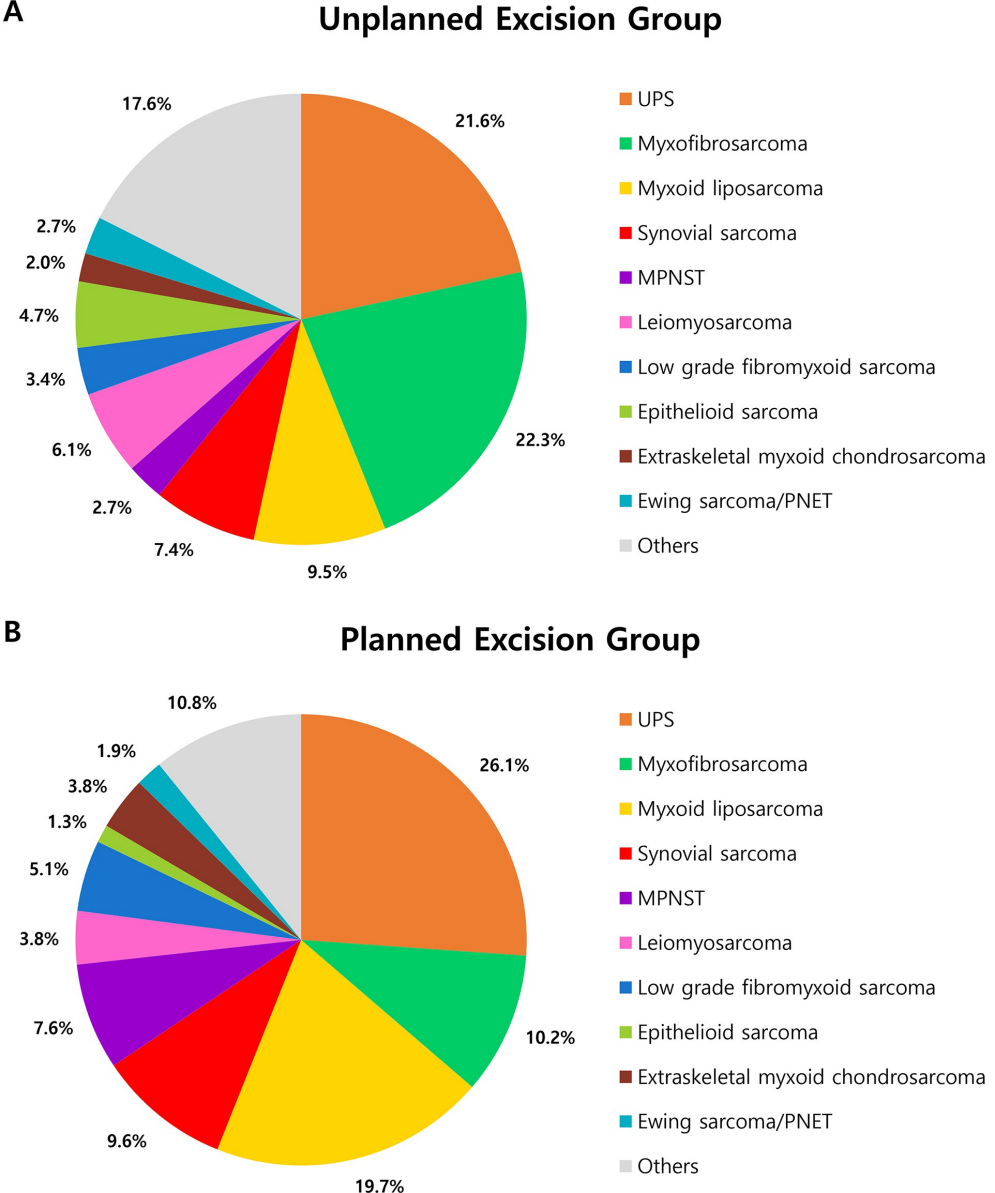
Distribution of histologic subtypes in **A** unplanned and **B** planned excision group. *UPS* undifferentiated pleomorphic sarcoma, *MPNST* malignant peripheral nerve sheath tumor, *PNET* primitive neuroectodermal tumor.

While undifferentiated pleomorphic sarcoma (UPS) was the most common subtype in all patients (n = 73, 23.9%), myxofibrosarcoma was most common in the unplanned excision group (n = 33, 22.3%). The proportion of myxofibrosarcomas that underwent unplanned excision was significantly higher than that of the overall STS (67.3% vs. 48.5%, p = 0.010). Contrastingly, the proportion of myxoid liposarcomas with unplanned excision was significantly lower compared to the overall STS (31.1% vs. 48.5%, p = 0.024). The proportion of unplanned excisions in the other histological subtypes did not differ significantly from the overall proportion of unplanned excisions (Table 1 and Fig 2).

**Table 1:** Detailed histological diagnosis and distribution of each histologic subtypes.

Histological Diagnosis	Total (n = 305)	Unplanned excision (n = 148) [48.5]	Planned excision (n = 157)	p value
UPS	73 (23.9)	32 (21.6) [43.8]	41 (26.1)	0.483
Myxofibrosarcoma	49 (16.1)	33 (22.3) [67.3]	16 (10.2)	0.010
Myxoid liposarcoma	45 (14.8)	14 (9.5) [31.1]	31 (19.8)	0.024
Synovial sarcoma	26 (8.5)	11 (7.4) [42.3]	15 (9.6)	0.568
MPNST	16 (5.3)	4 (2.7) [25]	12 (7.6)	0.079
Leiomyosarcoma	15 (4.9)	9 (6.1) [60]	6 (3.8)	0.444
Low grade fibromyxoid sarcoma	13 (4.3)	5 (3.4) [38.5]	8 (5.1)	0.583
Epithelioid sarcoma	9 (3.0)	7 (4.7) [77.8]	2 (1.3)	0.100
Extraskeletal myxoid chondrosarcoma	9 (3.0)	3 (2.0) [33.3]	6 (3.8)	0.510
Ewing sarcoma/PNET	7 (2.3)	4 (2.7) [57.1]	3 (1.9)	0.719
Others*	43 (14.1)	26 (17.6)	17 (10.8)	-

Unless otherwise indicated, data are presented as the number of patients, and numbers in parentheses indicate percentages of each histologic subtypes, numbers in brackets indicate proportion of unplanned excisions for each histological subtype. A one-sample proportion test was also conducted.

*Unspecified sarcoma (25), Pleomorphic liposarcoma (6), Clear cell sarcoma (5), Alveolar soft part sarcoma (2), Angiosarcoma (1), Dedifferentiated liposarcoma (1), Epithelioid hemangioendothelioma (1), Extraskeletal osteosarcoma (1), Malignant granular cell tumor (1).

*UPS*, undifferentiated pleomorphic sarcoma; *MPNST*, malignant peripheral nerve sheath tumor; *PNET*, primitive neuroectodermal tumor.

### Characteristics of STS

**Fig 3:**
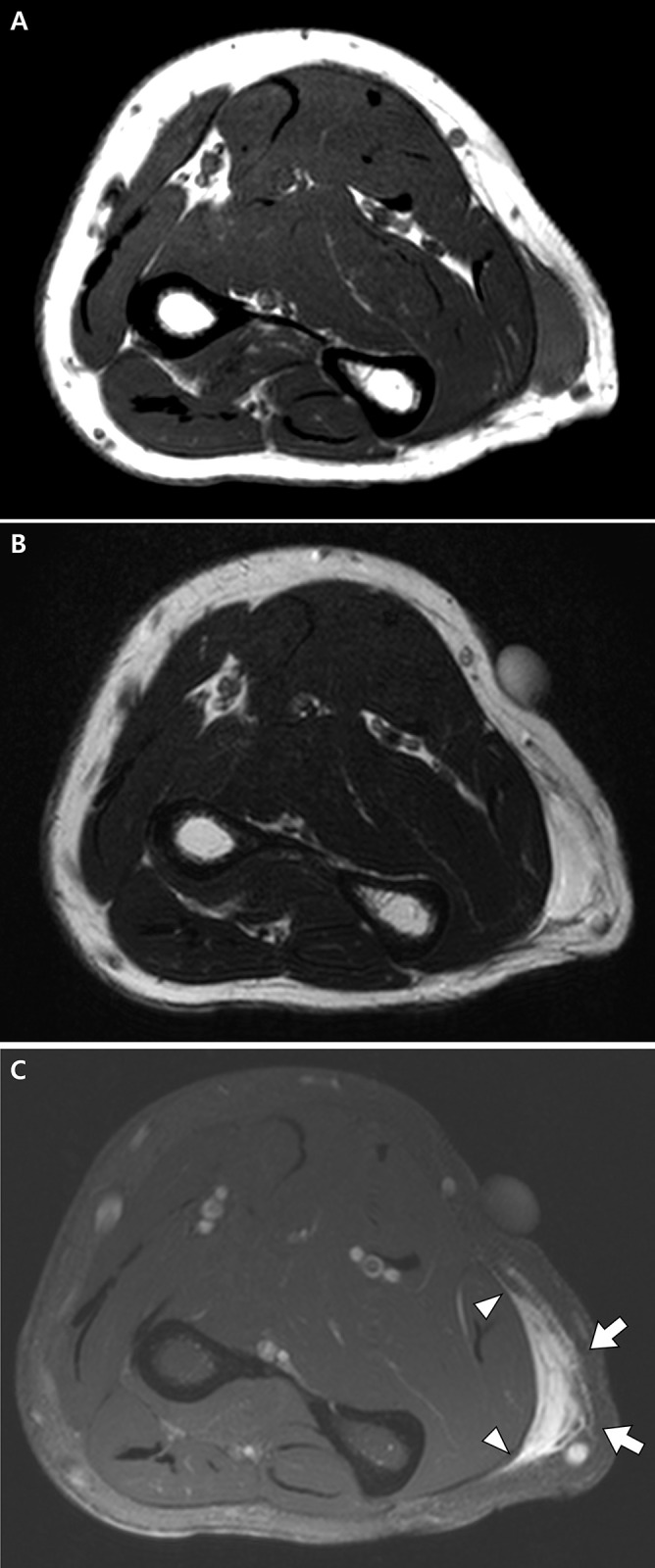
Magnetic resonance images of a 66-year-old man with myxofibrosarcoma before unplanned excision. **A** Axial T1- and **B** T2-weighted images of the right forearm show a 2.7 cm-sized mass in the subcutaneous fat layer. **C** Peritumoral abnormality is noted as an infiltrative enhancement along the superficial (arrows) and deep peripheral fascia (arrowheads) on axial fat-suppressed contrast-enhanced T1-weighted image.

**Fig 4:**
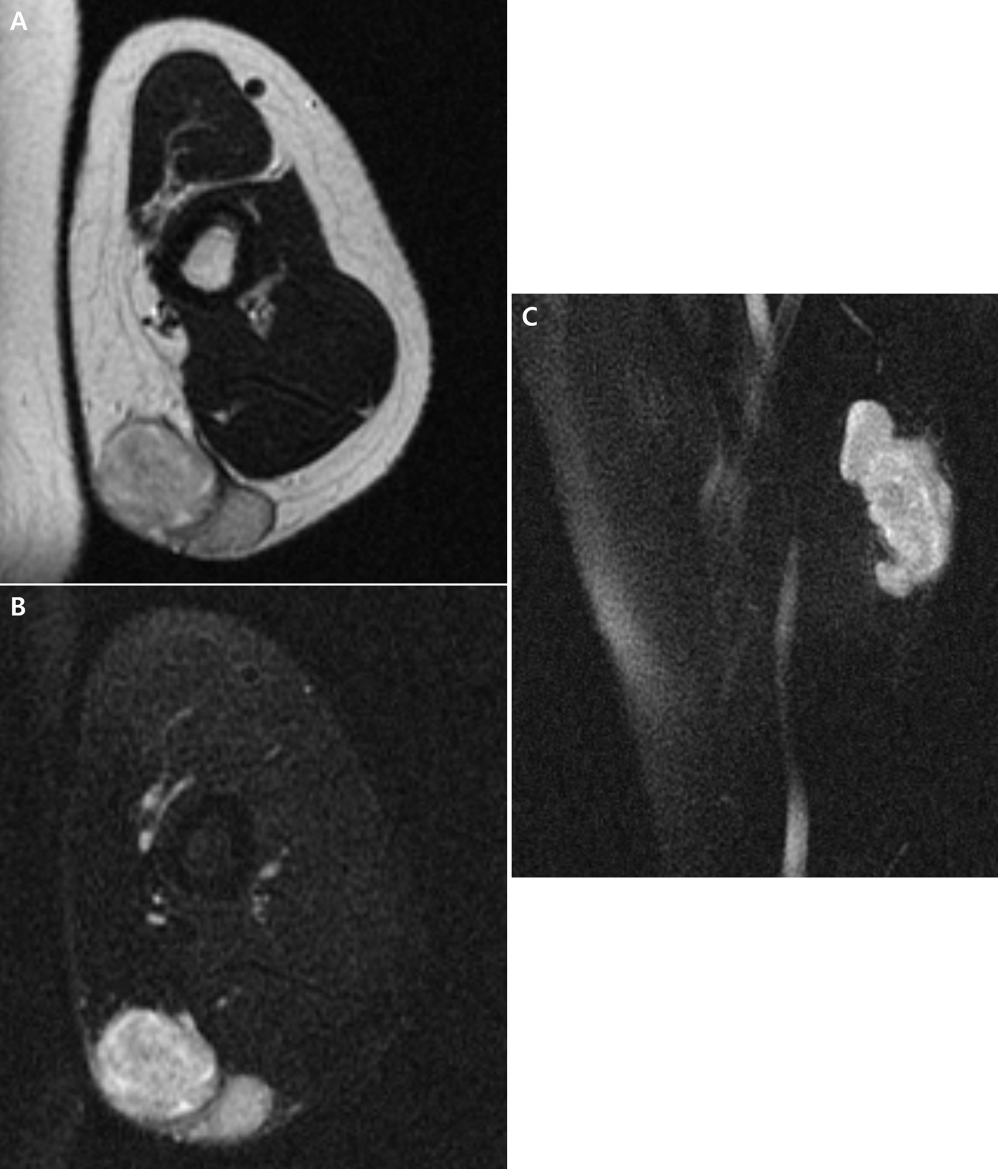
Magnetic resonance images of a 53-year-old woman with myxofibrosarcoma before unplanned excision. **A** Axial T2-, **B** axial fat-suppressed T2-, and **C** sagittal fat-suppressed T2-weighted images of left upper arm demonstrate a 4.4 cm-sized soft tissue mass in the subcutaneous fat layer with lobulated contour.

The tumor size was significantly smaller in the unplanned than in the planned excision group (median, 5.3 cm vs. 8.1 cm, p < 0.001). The tumor site was significantly different between the two groups (p = 0.002), with STS in the unplanned excision group being located more frequently in the upper (p = 0.015) than the lower (p < 0.001) extremities. In the unplanned excision group, the STS location was more frequently superficial compared to the planned excision group (57.5% vs. 20.6%, p < 0.001). Although less frequently than in the planned excision group (72.3%), peritumoral abnormalities were present in 50.0% in the unplanned excision group (p = 0.013) (Fig 3). Signal intensity heterogeneity was also less frequently observed in the unplanned excision group (17.5% vs. 44.5%, p = 0.003). No other clinical or MRI features showed significant difference between the two groups (p values > 0.05), including lobulated shape (Fig 4), which was noted in 70.0% of the unplanned excision group (Table 2).

**Table 2:** Characteristics of soft tissue sarcoma.

	Total (n = 305)	Unplanned excision (n = 148)	Planned excision (n = 157)	p value
Age (years)*	51 ± 16.4	52 ± 16.0	50 ± 17.0	0.465
Sex (men:women)^†^	179: 126	85: 63	94: 63	0.752
Tumor site^†^				0.002
Trunk	61 (20.0)	35 (23.6)	26 (16.6)	0.161
Upper extremity	75 (24.6)	46 (31.1)	29 (18.5)	0.015
Lower extremity	169 (55.4)	67 (45.3)	102 (65.0)	< 0.001
MRI features^‡^				
Size (cm)^§^	7.2 [4.6, 10.8]	5.3 [2.7, 7.3]	8.1 [5.0, 11.5]	< 0.001
Superficial location^†^	55 (28.2)	23 (57.5)	32 (20.6)	< 0.001
Lobulated shape^†^	141 (72.3)	28 (70.0)	113 (72.9)	0.867
Peritumoral abnormality^†^	132 (67.7)	20 (50.0)	112 (72.3)	0.013
Hemorrhage^†^^,^^ǁ^	49 (25.5)	7 (17.9)	42 (27.5)	0.313
Necrosis^†^^,^^¶^	59 (32.2)	7 (21.2)	52 (34.7)	0.197
Signal intensity heterogeneity^†^	76 (39.0)	7 (17.5)	69 (44.5)	0.003
Cyst-like appearance^†^	47 (24.1)	9 (22.5)	38 (24.5)	0.953
Bone involvement^#^	15 (7.7)	1 (2.5)	14 (9.0)	0.314
NVB encasement^#^	13 (6.7)	0 (0)	13 (8.4)	0.074

Unless otherwise indicated, data are presented as the number of patients, and numbers in parentheses indicate percentages.

*Two sample t-test. Data are presented as mean ± standard deviation.

^†^Chi-square test.

^‡^Available from 195 patients, including 40 in the unplanned excision group and 155 in the planned excision group.

^§^Mann-Whitney U test. Data are presented as the median [interquartile range].

^ǁ^Missing data on hemorrhage in three patients (one in the unplanned excision group and two in the planned excision group).

^¶^Missing data on necrosis in 12 patients (seven in the unplanned excision group and five in the planned excision group).

^#^Fisher’s exact test.

*NVB*, neurovascular bundle.

### Variables associated with unplanned excision of STS

Tumor size, site, location, peritumoral abnormality, and signal intensity heterogeneity were entered into the multivariable logistic regression analysis to identify variables associated with unplanned STS excision. Among these variables, only smaller tumor size (per 1 cm increase; adjusted OR, 0.87; 95% CI, 0.77–0.98; p = 0.025) and superficial location (adjusted OR, 3.48; 95% CI,1.57–7.72; p = 0.002) were independently associated with unplanned excision of STS (Table 3).

**Table 3:** Multivariable logistic regression analysis for unplanned excision.

	OR (95% CI)	p value
Size (per 1 cm increase)	0.87 (0.77–0.98)	0.025
Tumor site		
Trunk	Reference	-
Upper extremity	1.36 (0.36–5.22)	0.650
Lower extremity	1.14 (0.35–3.67)	0.824
Superficial location	3.48 (1.57–7.72)	0.002
Peritumoral abnormality	0.49 (0.22–1.10)	0.085
Signal intensity heterogeneity	0.47 (0.18–1.25)	0.128

Available from 195 patients, including 40 in the unplanned excision group and 155 in the planned excision group. Missing data in 110 patients, including 108 in the unplanned excision group and 2 in the planned excision group.

*OR*, odds ratio, *CI*, confidence interval.

## Discussion

Our study of patients with STS of the trunk and extremities revealed a difference in the distribution of histological subtypes between the unplanned and planned excision groups, with myxofibrosarcoma being frequently associated with unplanned excision. The investigation of preoperative MRI features in patients with STS who underwent unplanned excision was unique to our study. Although only small size and superficial location were independently associated with unplanned excision, our study suggests that a significant number of STS cases with unplanned excision exhibited suspicious MRI features [29]. These findings provide valuable insights into understanding unplanned excision of STS and may help prevent this undesirable surgical procedure, particularly for patients who have undergone preoperative MRI.

In a national registry study in Japan involving 8,761 patients, leiomyosarcoma, fibrosarcoma, and epithelioid sarcoma were frequently associated with unplanned excision, demonstrating a higher incidence of unplanned excision within these subtypes [14]. However, our findings revealed a contrasting pattern; myxofibrosarcoma was the most common subtype in the unplanned excision group, exhibiting a higher rate of unplanned excisions than in the overall STS population. Although speculative, the existence of a diverse and heterogeneous group of neoplasms comprising STS in our study may account for the observed differences.

Another explanation for this seeming contradiction is that our hospital is a tertiary referral center with a specialized center for sarcomas. The overall higher rate of unplanned excisions in our study (48.5%) compared to previous studies from national registries (11.3–36.5%) [11, 14, 15] could be attributed to our referral center status, as evidenced by a similar unplanned excision rate (53.3%) reported by another referral center [8].

Leiomyosarcomas are frequently associated with unplanned excision [13, 14]. However, superficial leiomyosarcoma is rare [30, 31] and is sometimes considered a distinct subtype from leiomyosarcoma of deep soft tissues [32, 33]. However, myxofibrosarcomas frequently present as superficially located tumors [34, 35] and constitute a significant proportion of superficial STS [25]. Thus, it seems reasonable to infer that the propensity of myxofibrosarcomas being in superficial locations may contribute to an increased risk of unplanned excision. The high frequency of myxofibrosarcoma observed in the unplanned excision group in our study also concurs with previous studies, indicating that superficial location of the STS was associated with unplanned excision [10, 12–15]. However, it should be noted that our findings may have been affected by selection bias, as this study was conducted at a single tertiary center with a smaller sample size compared to that of the national registry [14]. Thus, subsequent studies with larger patient populations are needed to generalize our results. Additionally, we observed a numerical association of unplanned excision in epithelioid sarcoma, with a high unplanned excision rate (77.8%), consistent with previous studies [13, 14]. Diagnosing epithelioid sarcoma is often challenging [36, 37] and may contribute to unplanned excision. However, this association was not significant, possibly because of small sample sizes.

Our analysis suggests that a significant number of unplanned excisions may occur due to the failure to recognize radiological findings associated with malignancy. In particular, it is noteworthy that about half of the patients in the unplanned excision group exhibited peritumoral abnormalities. Peritumoral abnormalities have been previously reported as indicators of malignancy in soft tissue tumors [17, 38] and are associated with high-grade STS [39]. For example, the “tail sign”—frequently observed in myxofibrosarcoma and characterized by thick fascial enhancement extending from the tumor margin—represents the fascial extension of the tumor [40, 41]. Noticing the lobulated shape, another potential indicator of STS [29], may also aid in preventing unplanned excision, given its high prevalence in the unplanned excision group. Thus, our findings imply that the incidence of unplanned excision could be reduced by considering the possibility of STS and the necessity of preoperative biopsy when these MRI features are detected, irrespective of the tumor size or location. Other MRI features, such as hemorrhage, necrosis, signal intensity heterogeneity, bone involvement, and neurovascular bundle involvement, were less frequently observed in the unplanned excision group. Notably, the frequency of signal intensity heterogeneity in this group was significantly lower than that in the planned excision group.

Regarding the independent significance of small size and superficial location, our results align with previous literature [8, 10, 12, 14, 15]. Our study further supports the previous notion that diagnosing small or superficially located soft tissue tumors poses challenges and carries a higher risk of unplanned excision, unlike large or deep-seated soft tissue lesions that are recommended for referral to specialized centers because of their potential for malignancy [2].

Besides analyzing histological subtypes, the novelty of our study lies in the initial evaluation and comparison of MRI features in patients with STS who underwent unplanned excision. However, this study had several limitations. First, it was a retrospective study conducted in a single tertiary referral center with a limited sample size. This may limit the generalizability of our findings to other settings, as discussed previously. Second, preoperative MRI was not available for a larger proportion of patients in the unplanned than in the planned excision group, potentially introducing further selection bias and affecting the statistical power. However, this limitation seems unavoidable, since preoperative imaging is omitted more frequently in cases of STS that undergo unplanned excision, with reported rate of 73.7% [15]. In this regard, additional efforts are needed to minimize the occurrence of unplanned excision without appropriate preoperative imaging. While analyzing the characteristics of the patients within the unplanned excision group who did not receive preoperative imaging may contribute to such efforts, it was not feasible due to the lack of precise data regarding whether the absence of MRI in these patients resulted from the non-performance of MRI or other factors. Third, discerning MRI features in small tumors can be challenging than in larger tumors. This may have affected the outcomes of our study, considering that tumors in the unplanned excision group tended to be smaller compared to those in the planned excision group. Fourth, there was limited data on the preoperative diagnoses in patients who underwent unplanned excision. Finally, it is possible that the patient characteristics of STS in the unplanned excision group may differ from those in the planned excision group, given that most patients in the unplanned excision group were referred to our hospital after being excised at an outside facility. Based on our findings, further studies with larger sample sizes and multicenter comparisons are required.

## Conclusions

In conclusion, myxofibrosarcoma is associated with a high frequency of unplanned STS excision in the trunk and extremities. Small and superficial STS remain diagnostic challenges and are independently associated with unplanned excision. However, a considerable proportion of STS cases that undergo unplanned excision display MRI features that could raise suspicion of STS, such as peritumoral abnormality and lobulated shape. By carefully examining preoperative MRI, we believe that identifying these findings and considering preoperative biopsy could significantly reduce this unintended surgical procedure and improve clinical outcomes in STS. Future research that thoroughly examines the clinical characteristics of patients with STS and aims to prevent the omission of preoperative imaging could complement our findings focused on radiological aspects and ultimately reduce the incidence of unplanned excisions of STS.

## Supporting information

S1 Fig Magnetic resonance images of a 53-year-old woman with leiomyosarcoma before unplanned excision. **A** Axial T1- and **B** T2-weighted images of the right thigh show a 3.7 cm-sized mass located superficial to the deep peripheral fascia. The mass shows lobulated contour.
(TIFF)

S2 Fig Magnetic resonance images of a 47-year-old man with myxoid liposarcoma before planned excision. **A** Axial T1-weighted image and **B** axial fat-suppressed T2 weighted image show weighted image of right lower leg show 5.1 cm-sized tumor with lobulated contour. The tumor showed bright signal intensity on T2-weighted image and low signal intensity on T1-weighted image, defined as cyst-like appearance.
(TIFF)

S3 Fig Magnetic resonance images of a 21-year-old man with clear cell sarcoma before planned excision. **A** Axial T1-weighted image of right ankle shows 5.1 cm-sized tumor with hemorrhage (straight arrow) appearing as foci of high signal intensity on T1-weighted image that does not show signal drop on fat-suppressed images (not shown). **B** Axial T2-weighted image shows signal intensity heterogeneity. **C** Coronal T1-weighted image and **D** coronal fat-suppressed contrast-enhanced T1-weighted image show bone involvement with change in cortical and medullary signal intensity and cortical destruction (curved arrow). **E** Coronal fat-suppressed contrast-enhanced T1-weighted image shows necrosis (asterisk), showing non-enhancing necrotic fluid with an irregular margin and rim enhancement. Peritumoral abnormality is noted as areas of infiltrative enhancement (arrowheads).
(TIFF)

S4 Fig Magnetic resonance images of a 48-year-old man with undifferentiated liposarcoma before planned excision. Axial T2-weighted image of right upper arm shows 5.5 cm-sized tumor with neurovascular bundle involvement. The tumor abutment of the median nerve (straight arrow) exceeds 180 degrees. Adjacent brachial artery (asterisk), brachial vein (curved arrow) and ulnar nerve (arrowhead) are also noted.
(TIFF)

S1 File Supporting data. Excel file contains dataset used and analysed during current study.
(XLSX)
