# Supplementary figures and images for: Clinical and radiologic features of soft tissue sarcoma in trunk and extremities that underwent unplanned excision
Source: PLoS One. 2024 Dec 5;19(12):e0311300. doi: 10.1371/journal.pone.0311300 (PMC11620571; doi:10.1371/journal.pone.0311300)

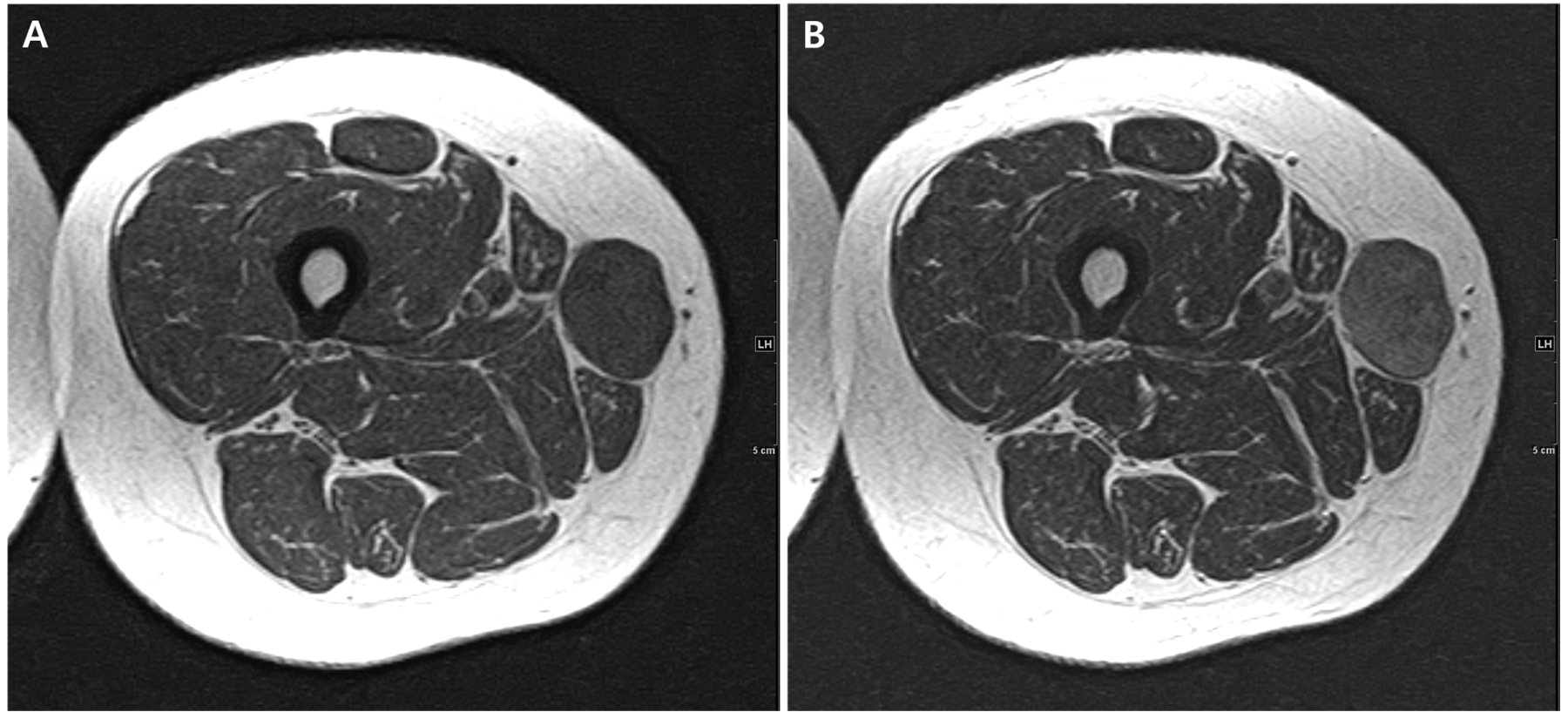

Supplement: S1 Fig — A Axial T1- and B T2-weighted images of the right thigh show a 3.7 cm-sized mass located superficial to the deep peripheral fascia. The mass shows lobulated contour. (TIFF) [file pone.0311300.s001.tiff]

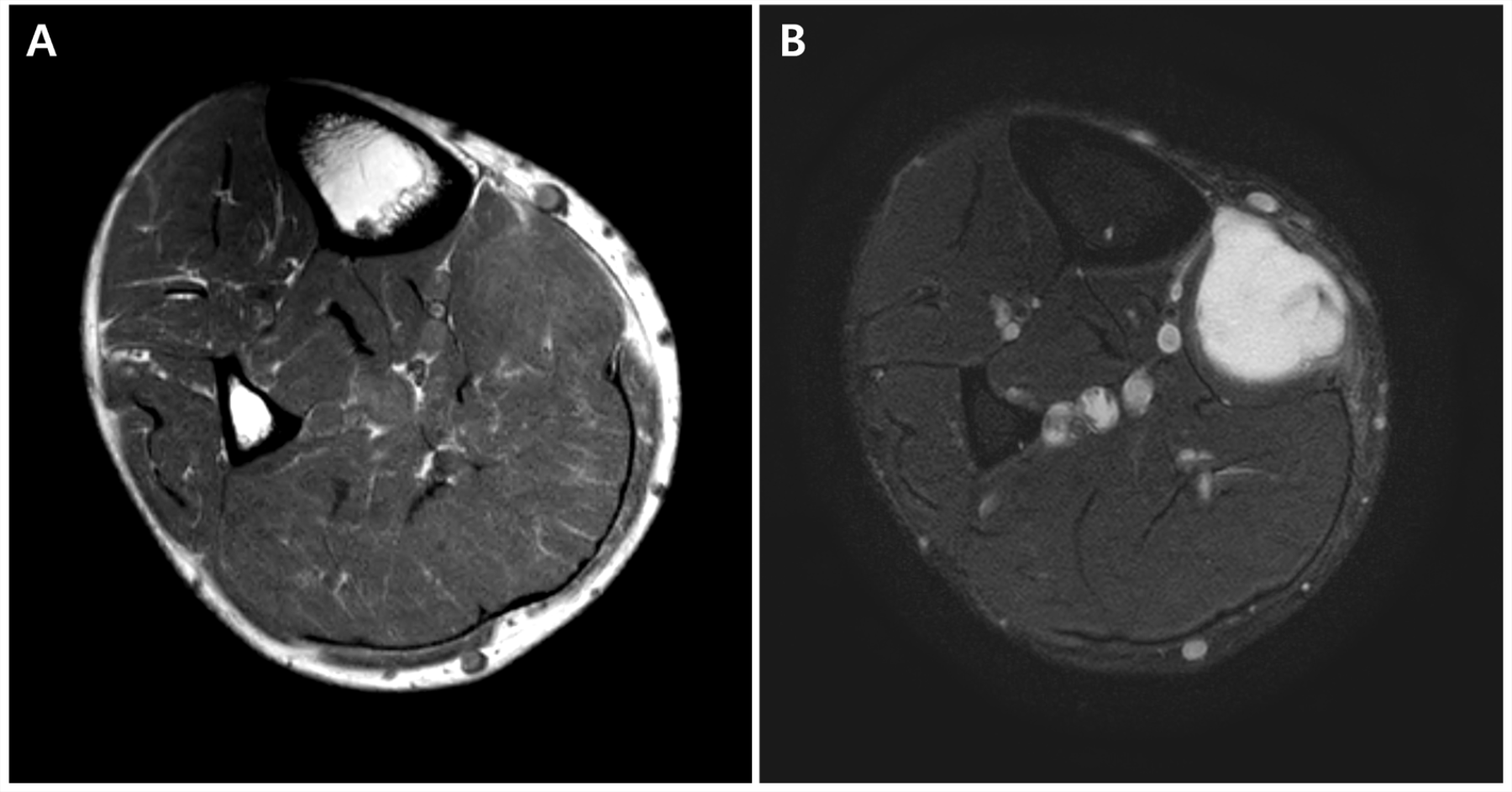

Supplement: S2 Fig — A Axial T1-weighted image and B axial fat-suppressed T2 weighted image show weighted image of right lower leg show 5.1 cm-sized tumor with lobulated contour. The tumor showed bright signal intensity on T2-weighted image and low signal intensity on T1-weighted image, defined as cyst-like appearance. (TIFF) [file pone.0311300.s002.tiff]

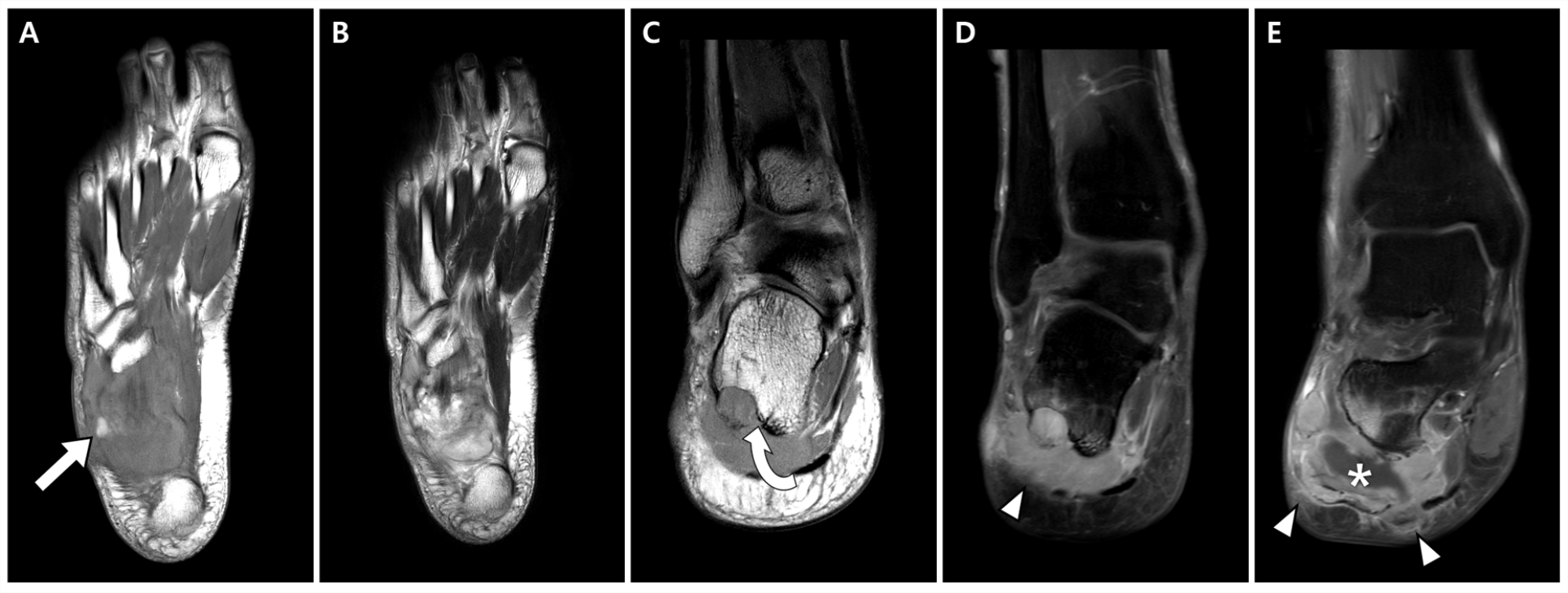

Supplement: S3 Fig — A Axial T1-weighted image of right ankle shows 5.1 cm-sized tumor with hemorrhage (straight arrow) appearing as foci of high signal intensity on T1-weighted image that does not show signal drop on fat-suppressed images (not shown). B Axial T2-weighted image shows signal intensity heterogeneity. C Coronal T1-weighted image and D coronal fat-suppressed contrast-enhanced T1-weighted image show bone involvement with change in cortical and medullary signal intensity and cortical destruction (curved arrow). E Coronal fat-suppressed contrast-enhanced T1-weighted image shows necrosis (asterisk), showing non-enhancing necrotic fluid with an irregular margin and rim enhancement. Peritumoral abnormality is noted as areas of infiltrative enhancement (arrowheads). (TIFF) [file pone.0311300.s003.tiff]

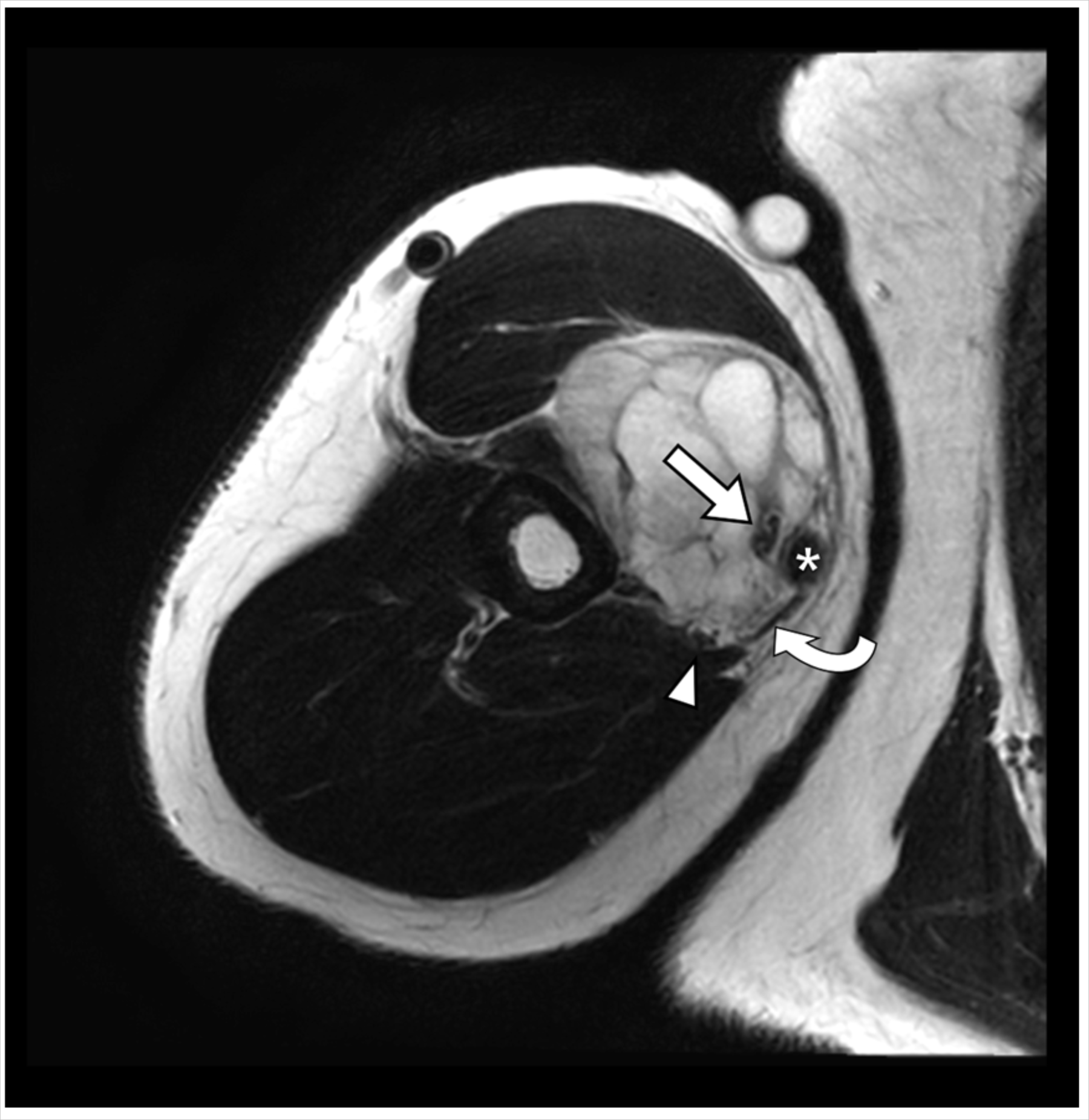

Supplement: S4 Fig — Axial T2-weighted image of right upper arm shows 5.5 cm-sized tumor with neurovascular bundle involvement. The tumor abutment of the median nerve (straight arrow) exceeds 180 degrees. Adjacent brachial artery (asterisk), brachial vein (curved arrow) and ulnar nerve (arrowhead) are also noted. (TIFF) [file pone.0311300.s004.tiff]
